# (*Z*)-4-(2-Naphthyl­amino)­pent-3-en-2-one

**DOI:** 10.1107/S1600536811024494

**Published:** 2011-06-25

**Authors:** Mohamed Anoir Harrad, Brahim Boualy, Abdelghani Oudahmane, Daniel Avignant, Corrado Rizzoli

**Affiliations:** a Laboratoire de Chimie de Coordination, Faculté des Sciences-Semlalia, BP 2390, 40001 Marrakech, Morocco; b Université Blaise Pascal, Laboratoire des Matèriaux Inorganiques, UMR CNRS 6002, 24 Avenue des Landais, 63177 Aubiére, France; c Dipartimento di Chimica Generale ed Inorganica, Chimica Analitica, Chimica Fisica, Universitá degli Studi di Parma, Viale G. P. Usberti 17/A, I-43124 Parma, Italy

## Abstract

The title compound, C_15_H_15_NO, which was synthesized under solvent-free conditions by the reaction of acetoacetone and 2-naphthyl­amine, adopts a *Z* conformation about the C=C bond. The enamine–ketone fragment is approximately planar [maximum deviation = 0.026 (3) Å] and forms a dihedral angle of 39.78 (3)° with the naphthalene ring system. An intra­molecular N—H⋯O hydrogen bond is observed.

## Related literature

For our studies on the synthesis of β-enamino­nes and β-enamino esters, see: Harrad *et al.* (2010 ▶, 2011 ▶). For related structures, see: Shaheen *et al.* (2006 ▶); Arıcı *et al.* (1999 ▶).
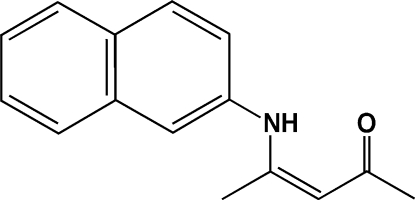


## Experimental

### 

#### Crystal data

C_15_H_15_NO
*M* *_r_* = 225.28
Orthorhombic, 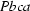

*a* = 11.2417 (18) Å
*b* = 8.2532 (10) Å
*c* = 26.570 (4) Å
*V* = 2465.2 (6) Å^3^
*Z* = 8
Mo *K*α radiation
μ = 0.08 mm^−1^
*T* = 296 K
0.48 × 0.34 × 0.12 mm

#### Data collection

Bruker APEXII CCD diffractometer
Absorption correction: multi-scan (*SADABS*, Bruker, 2008 ▶) *T*_min_ = 0.660, *T*_max_ = 0.746
9535 measured reflections
2221 independent reflections
1179 reflections with *I* > 2σ(*I*)
*R*_int_ = 0.057

#### Refinement

*R*[*F* ^2^ > 2σ(*F* ^2^)] = 0.050
*wR*(*F* ^2^) = 0.142
*S* = 0.94
2221 reflections
159 parameters
H atoms treated by a mixture of independent and constrained refinement
Δρ_max_ = 0.19 e Å^−3^
Δρ_min_ = −0.15 e Å^−3^

### 

Data collection: *APEX2* (Bruker, 2008 ▶); cell refinement: *SAINT* (Bruker, 2008 ▶); data reduction: *SAINT*; program(s) used to solve structure: *SHELXS97* (Sheldrick, 2008 ▶); program(s) used to refine structure: *SHELXL97* (Sheldrick, 2008 ▶); molecular graphics: *ORTEP-3 for Windows* (Farrugia, 1997 ▶) and *SCHAKAL97* (Keller, 1997 ▶); software used to prepare material for publication: *SHELXL97* and *PARST95* (Nardelli, 1995 ▶).

## Supplementary Material

Crystal structure: contains datablock(s) global, I. DOI: 10.1107/S1600536811024494/ng5187sup1.cif

Structure factors: contains datablock(s) I. DOI: 10.1107/S1600536811024494/ng5187Isup2.hkl

Supplementary material file. DOI: 10.1107/S1600536811024494/ng5187Isup3.cml

Additional supplementary materials:  crystallographic information; 3D view; checkCIF report

## Figures and Tables

**Table 1:** Hydrogen-bond geometry (Å, °)

*D*—H⋯*A*	*D*—H	H⋯*A*	*D*⋯*A*	*D*—H⋯*A*
N1—H1*N*⋯O1	0.92 (2)	1.85 (2)	2.657 (2)	144 (2)

**Fig. 1.**
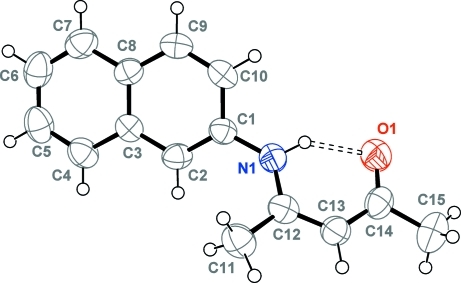
The molecular structure of the title compound, with displacement ellipsoids drawn at the 50% probability level. The intramolecular hydrogen bond is shown as a dashed line.

**Fig. 2.**
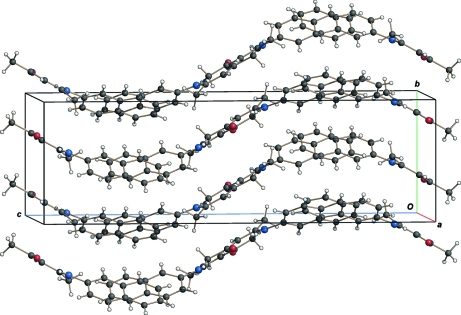
Packing diagram of the title compound approximately viewed along the a axis.

## supplementary crystallographic information

### Comment

β-Enaminones and β-enaminoesters are useful precursors for the preparation of biologically active compounds such as β-enamino acids and γ-enamino alcohols, and many synthetic methods have been developed for the preparation of these compounds. As a continuation of our work on the synthesis and characterization of new β-enamino compounds (Harrad *et al.*, 2010, 2011), we describe herein the crystal structure of title compound.

The title compound (Fig. 1) crystallizes in the keto-enamine form, as indicated by values of the C14═O1 and C13–C14 bond length of 1.252 (3) and 1.410 (3) Å, respectively. The bond lengths observed within the C13–C12–N1 chain (C12–C13 = 1.375 (3) Å; N1–C12 = 1.353 (3) Å) suggest some degree of electron delocalization of the imino and alkene double bonds. The molecule assumes a *Z* conformation about the C12═C13 bond. An *S*(6) ring motif is formed due to an intramolecular N—H···O hydrogen bond (Table 1). The enamino-ketone fragment (N1/C12/*c*13/C14/O1) is approximately planar (maximum deviation 0.026 (3) Å for atom C14) and is twisted by 39.78 (3)° with respect to the naphthalene ring. This value is comparable with those of 32.06 (9) and 44.71 (7)° found in (*Z*)-4-anilinopent-3-en-2-one (Shaheen *et al.*, 2006) and 4-chloro-2-(4-oxopent-2-en-2-ylamino)phenol (Arıcı *et al.*, 1999), respectively. The crystal packing (Fig. 2) is governed only by van der Waals interactions. No C—H···π or π···π interactions are observed.

### Experimental

A mixture of acetoacetone (5 mmol), 2-naphthylamine (5 mmol) and Ca(CF_3_CO_2_)_2_ (0.05 mmol) was stirred at room temperature for 1 h under solvent-free conditions. After completion of the reaction, the mixture was diluted with H_2_O (10 ml), extracted with EtOAc (2 × 10 ml) and dried over Na_2_SO_4_. The title compound was isolated as a white powder by column chromatography on silica gel using ethyl acetate/*n*-hexane (1:1 *v*/*v*) as eluent (yield 62%; m. p.= 395 K). Colourless single crystals suitable for X-ray analysis were obtained by slow evaporation at room temperature of an *n*-hexane solution. ^1^H NMR (CDCl_3_, 300 MHz) δ: 1.9 (s; 3H), 2.2 (s, 3H), 3.1 (s, 1*H*); 7.2–7.7 (m, 7*H*, Ar), 12.6 (bs, 1*H*, HN); ^13^C NMR (CDCl_3_, 75 MHz) δ: 19.96, 30.53, 97.04, 127.95, 130.10, 132.50, 135.14, 126.61, 125.23; 124.63, 122.81; 120.58; 159.25, 195.23. EIMS (m/*z*) 226.1 (*M*^+^). HRMS calcd for C_15_H_15_NO: 225.1154; found 225.1163.

### Refinement

The amine H atom was located in a difference Fourier map and refined freely. All other H atoms were fixed geometrically and treated as riding, with C–H = 0.93–0.96 Å, and with *U*_iso_(H) = 1.2 *U*_eq_(C) or 1.5 *U*_eq_(C) for methyl H atoms. A rotating group model was used for the methyl groups. Four low-angle reflections [2 0 0 (θ = 3.62°), 1 1 1 (θ = 3.16°), 1 0 2 (θ = 2.37°) and 1 1 2 (θ = 3.42°)] were omitted from the final cycles of refinement because their observed intensities were much lower than the calculated values as a result of being affected by the beam stop.

### Crystal data

**Table:**

C_15_H_15_NO	*F*(000) = 960
*M**_r_* = 225.28	*D*_x_ = 1.214 Mg m^−^^3^
Orthorhombic, *P**b**c**a*	Mo *K*α radiation, λ = 0.71073 Å
Hall symbol: -P 2ac 2ab	Cell parameters from 1094 reflections
*a* = 11.2417 (18) Å	θ = 3.1–19.4°
*b* = 8.2532 (10) Å	µ = 0.08 mm^−^^1^
*c* = 26.570 (4) Å	*T* = 296 K
*V* = 2465.2 (6) Å^3^	Plate, colourless
*Z* = 8	0.48 × 0.34 × 0.12 mm

### Data collection

**Table:**

Bruker APEXII CCD diffractometer	2221 independent reflections
Radiation source: fine-focus sealed tube	1179 reflections with *I* > 2σ(*I*)
graphite	*R*_int_ = 0.057
ω and φ scans	θ_max_ = 25.3°, θ_min_ = 1.5°
Absorption correction: multi-scan (*SADABS*, Bruker, 2008)	*h* = −13→8
*T*_min_ = 0.660, *T*_max_ = 0.746	*k* = −9→9
9535 measured reflections	*l* = −31→31

### Refinement

**Table:**

Refinement on *F*^2^	Secondary atom site location: difference Fourier map
Least-squares matrix: full	Hydrogen site location: inferred from neighbouring sites
*R*[*F*^2^ > 2σ(*F*^2^)] = 0.050	H atoms treated by a mixture of independent and constrained refinement
*wR*(*F*^2^) = 0.142	*w* = 1/[σ^2^(*F*_o_^2^) + (0.0749*P*)^2^] where *P* = (*F*_o_^2^ + 2*F*_c_^2^)/3
*S* = 0.94	(Δ/σ)_max_ < 0.001
2221 reflections	Δρ_max_ = 0.19 e Å^−^^3^
159 parameters	Δρ_min_ = −0.15 e Å^−^^3^
0 restraints	Extinction correction: *SHELXL97* (Sheldrick, 2008)
Primary atom site location: structure-invariant direct methods	Extinction coefficient: 0.011 (2)

### Special details

**Table:**

Refinement. Refinement of *F*^2^ against ALL reflections. The weighted *R*-factor *wR* and goodness of fit *S* are based on *F*^2^, conventional *R*-factors *R* are based on *F*, with *F* set to zero for negative *F*^2^. The threshold expression of *F*^2^ > σ(*F*^2^) is used only for calculating *R*-factors(gt) *etc*. and is not relevant to the choice of reflections for refinement. *R*-factors based on *F*^2^ are statistically about twice as large as those based on *F*, and *R*- factors based on ALL data will be even larger.

### Fractional atomic coordinates and isotropic or equivalent isotropic displacement parameters (Å^2^)

**Table:**

	*x*	*y*	*z*	*U*_iso_*/*U*_eq_	
O1	0.37331 (17)	0.7158 (2)	0.48799 (6)	0.0702 (6)	
N1	0.3060 (2)	0.5679 (2)	0.57214 (7)	0.0544 (6)	
H1N	0.359 (2)	0.602 (3)	0.5479 (9)	0.081 (9)*	
C1	0.3453 (2)	0.5091 (3)	0.61903 (8)	0.0468 (6)	
C2	0.2893 (2)	0.5426 (3)	0.66352 (8)	0.0526 (6)	
H2	0.2212	0.6066	0.6633	0.063*	
C3	0.3326 (2)	0.4819 (2)	0.70984 (8)	0.0465 (6)	
C4	0.2751 (2)	0.5120 (3)	0.75613 (9)	0.0616 (7)	
H4	0.2051	0.5722	0.7566	0.074*	
C5	0.3207 (3)	0.4541 (3)	0.80021 (9)	0.0713 (8)	
H5	0.2816	0.4749	0.8304	0.086*	
C6	0.4261 (3)	0.3634 (3)	0.80025 (10)	0.0714 (8)	
H6	0.4565	0.3241	0.8304	0.086*	
C7	0.4840 (2)	0.3327 (3)	0.75657 (10)	0.0646 (7)	
H7	0.5540	0.2727	0.7571	0.077*	
C8	0.4395 (2)	0.3907 (2)	0.71006 (8)	0.0489 (6)	
C9	0.4972 (2)	0.3633 (3)	0.66390 (9)	0.0573 (7)	
H9	0.5681	0.3050	0.6635	0.069*	
C10	0.4518 (2)	0.4200 (2)	0.61962 (8)	0.0546 (6)	
H10	0.4917	0.3996	0.5896	0.065*	
C11	0.0924 (2)	0.5082 (3)	0.58249 (9)	0.0661 (7)	
H11A	0.0820	0.5612	0.6143	0.099*	
H11B	0.1095	0.3956	0.5879	0.099*	
H11C	0.0209	0.5183	0.5630	0.099*	
C12	0.1937 (2)	0.5854 (3)	0.55465 (8)	0.0517 (6)	
C13	0.1742 (2)	0.6647 (3)	0.50990 (8)	0.0563 (7)	
H13	0.0958	0.6769	0.4993	0.068*	
C14	0.2644 (3)	0.7286 (3)	0.47888 (9)	0.0574 (7)	
C15	0.2277 (3)	0.8137 (3)	0.43102 (9)	0.0835 (9)	
H15A	0.2948	0.8702	0.4172	0.125*	
H15B	0.1654	0.8897	0.4383	0.125*	
H15C	0.1995	0.7353	0.4071	0.125*	

### Atomic displacement parameters (Å^2^)

**Table:**

	*U*^11^	*U*^22^	*U*^33^	*U*^12^	*U*^13^	*U*^23^
O1	0.0617 (13)	0.0837 (12)	0.0651 (11)	−0.0036 (10)	0.0047 (10)	0.0057 (9)
N1	0.0499 (14)	0.0651 (12)	0.0481 (12)	−0.0006 (11)	0.0026 (12)	0.0017 (10)
C1	0.0438 (15)	0.0486 (12)	0.0481 (15)	0.0001 (11)	0.0000 (11)	−0.0053 (11)
C2	0.0497 (16)	0.0521 (14)	0.0560 (15)	0.0084 (11)	−0.0038 (12)	−0.0073 (11)
C3	0.0450 (15)	0.0457 (12)	0.0488 (14)	−0.0069 (11)	0.0007 (12)	−0.0071 (10)
C4	0.0576 (17)	0.0708 (15)	0.0563 (16)	−0.0027 (13)	0.0031 (14)	−0.0120 (12)
C5	0.076 (2)	0.0848 (19)	0.0529 (17)	−0.0202 (17)	0.0030 (16)	−0.0073 (13)
C6	0.073 (2)	0.0849 (18)	0.0559 (17)	−0.0205 (16)	−0.0149 (16)	0.0106 (14)
C7	0.0530 (16)	0.0665 (15)	0.0742 (18)	−0.0044 (12)	−0.0118 (15)	0.0069 (13)
C8	0.0447 (15)	0.0463 (12)	0.0556 (14)	−0.0030 (11)	−0.0065 (12)	−0.0001 (11)
C9	0.0422 (15)	0.0607 (14)	0.0690 (17)	0.0057 (11)	0.0001 (13)	−0.0026 (13)
C10	0.0479 (16)	0.0594 (14)	0.0563 (15)	−0.0002 (12)	0.0105 (12)	−0.0080 (12)
C11	0.0552 (17)	0.0758 (15)	0.0673 (16)	−0.0156 (14)	0.0013 (13)	−0.0088 (13)
C12	0.0499 (16)	0.0519 (13)	0.0532 (14)	−0.0027 (11)	−0.0009 (13)	−0.0139 (11)
C13	0.0505 (16)	0.0678 (15)	0.0506 (14)	0.0000 (13)	−0.0048 (13)	−0.0079 (12)
C14	0.072 (2)	0.0537 (14)	0.0467 (14)	0.0015 (13)	−0.0084 (15)	−0.0070 (11)
C15	0.110 (3)	0.0807 (19)	0.0595 (16)	−0.0019 (16)	−0.0137 (16)	0.0079 (13)

### Geometric parameters (Å, °)

**Table:**

O1—C14	1.252 (3)	C7—H7	0.9300
N1—C12	1.353 (3)	C8—C9	1.406 (3)
N1—C1	1.408 (3)	C9—C10	1.365 (3)
N1—H1N	0.92 (3)	C9—H9	0.9300
C1—C2	1.368 (3)	C10—H10	0.9300
C1—C10	1.405 (3)	C11—C12	1.500 (3)
C2—C3	1.415 (3)	C11—H11A	0.9600
C2—H2	0.9300	C11—H11B	0.9600
C3—C4	1.412 (3)	C11—H11C	0.9600
C3—C8	1.418 (3)	C12—C13	1.375 (3)
C4—C5	1.365 (3)	C13—C14	1.410 (3)
C4—H4	0.9300	C13—H13	0.9300
C5—C6	1.401 (4)	C14—C15	1.510 (3)
C5—H5	0.9300	C15—H15A	0.9600
C6—C7	1.354 (3)	C15—H15B	0.9600
C6—H6	0.9300	C15—H15C	0.9600
C7—C8	1.416 (3)		
			
C12—N1—C1	129.3 (2)	C10—C9—C8	121.6 (2)
C12—N1—H1N	109.5 (16)	C10—C9—H9	119.2
C1—N1—H1N	121.1 (16)	C8—C9—H9	119.2
C2—C1—C10	119.2 (2)	C9—C10—C1	120.5 (2)
C2—C1—N1	123.4 (2)	C9—C10—H10	119.7
C10—C1—N1	117.24 (19)	C1—C10—H10	119.7
C1—C2—C3	121.4 (2)	C12—C11—H11A	109.5
C1—C2—H2	119.3	C12—C11—H11B	109.5
C3—C2—H2	119.3	H11A—C11—H11B	109.5
C4—C3—C2	122.5 (2)	C12—C11—H11C	109.5
C4—C3—C8	118.6 (2)	H11A—C11—H11C	109.5
C2—C3—C8	118.90 (19)	H11B—C11—H11C	109.5
C5—C4—C3	120.9 (2)	N1—C12—C13	119.8 (2)
C5—C4—H4	119.5	N1—C12—C11	119.6 (2)
C3—C4—H4	119.5	C13—C12—C11	120.5 (2)
C4—C5—C6	120.4 (2)	C12—C13—C14	124.7 (2)
C4—C5—H5	119.8	C12—C13—H13	117.7
C6—C5—H5	119.8	C14—C13—H13	117.7
C7—C6—C5	120.3 (2)	O1—C14—C13	124.0 (2)
C7—C6—H6	119.8	O1—C14—C15	118.0 (2)
C5—C6—H6	119.8	C13—C14—C15	118.0 (2)
C6—C7—C8	121.0 (2)	C14—C15—H15A	109.5
C6—C7—H7	119.5	C14—C15—H15B	109.5
C8—C7—H7	119.5	H15A—C15—H15B	109.5
C9—C8—C7	122.9 (2)	C14—C15—H15C	109.5
C9—C8—C3	118.25 (19)	H15A—C15—H15C	109.5
C7—C8—C3	118.8 (2)	H15B—C15—H15C	109.5

### Hydrogen-bond geometry (Å, °)

**Table:**

*D*—H···*A*	*D*—H	H···*A*	*D*···*A*	*D*—H···*A*
N1—H1N···O1	0.92 (2)	1.85 (2)	2.657 (2)	144 (2)
